# The Effects of Increased Body Temperature on Motor Control during Golf Putting

**DOI:** 10.3389/fpsyg.2016.01246

**Published:** 2016-08-31

**Authors:** John F. Mathers, Madeleine A. Grealy

**Affiliations:** ^1^School of Sport, University of StirlingStirling, UK; ^2^School of Psychological Sciences and Health, University of StrathclydeGlasgow, UK

**Keywords:** scaling, increased body temperature, motor skill, golf putting, movement kinematics

## Abstract

This study investigated the effect of increased core temperature on the performance outcome and movement kinematics of elite golfers during a golf putting task. The study aimed to examine individual differences in the extent to which increased temperature influenced the rate of putting success, whether increased temperature speeded up the timing of the putting downswing and whether elite golfers changed their movement kinematics during times of thermal stress. Six participants performed 20 putts to each of four putt distances (1, 2, 3, and 4 m) under normal temperature conditions and when core body temperature was increased. There was no significant difference in the number of successful putts between the two temperature conditions, but there was an increase in putterhead velocity at ball impact on successful putts to distances of 1 and 4 m when temperature was elevated. This reflected an increase in swing amplitude rather than a reduction in swing duration as hypothesized. There were individual differences in the motor control response to thermal stress as three of the golfers changed the kinematic parameters used to scale their putting movements to achieve putts of different distances at elevated temperatures. Theoretical implications for these findings and the practical implications for elite golfers and future research are discussed.

## Introduction

Success in golf putting requires the golfer to perceive the speed of the putting surface, the degree of slope to be negotiated on the green, then strike the ball with a force and direction that sends it toward the hole with the pre-determined velocity (Pelz, 2000; Penner, 2002; Hume et al., 2005). The way in which golfers adjust the force applied to the ball to ‘hole’ putts of different distances was investigated by Craig et al. (2000) who used a series of mathematical equations to demonstrate that the distance traveled by the ball is proportional to the squared velocity of the putterhead at impact (*V*_c_^2^):

$$V_{c}^{2}=(2\lambda M_{c}/F)D^{2}(1/T^{2}){\left(P_{t}/k\right)}^{2}{\left(1-P_{t}^{2}\right)}^{(2/k)-2}$$

where *M*c is the effective mass of the club-body system, *D* is the amplitude of the downswing, *P*_t_ is the proportion of the swing duration before the ball is struck, *T* the duration of the swing, *k* the point at which peak velocity occurs in the swing, and λ is a constant.

Craig et al. (2000) suggested that the most efficient method of scaling the putting action to achieve different putt distances was by changing the amplitude of the downswing (*D*^2^) whilst keeping the other variables in this equation constant, a method that appeals to the simplicity principle advocated by golf instruction texts (Owens and Bunker, 1989; Pelz, 2000), and preserves the temporal relationships of intra-movement segments within movement scaling (Schmidt, 2003). Mathers and Grealy (2014) used a motion capture system to record the movement kinematics used by six elite golfers when putting to assess whether they adopted a method of adjusting swing amplitude as suggested by Craig et al. (2000). They found that within these six golfers two different methods were used to achieve putts of various distances; one method was to systematically vary the swing amplitude and duration, and the other was to systematically vary amplitude, duration and the proportion of the swing duration before the ball was struck. Whilst this was not predicted by Craig et al. (2000) these individual differences were not unexpected given the variability of the neuro-physiological system and the range of possible strategies used to hole short to medium length putts (i.e., a higher impact velocity with less allowance for the natural contours of the green, or a slower impact velocity that allows the ball to embrace the slope of the green into the hole) (Newell and Corcos, 1993; Pelz, 2000). Mathers and Grealy (2014) also examined the effect of fatigue (induced by treadmill walking) on the methods of scaling and found that three of the six golfers showed significant changes in how they controlled the putterhead when they reported feeling fatigued. These fatigue-related kinematic changes were evident in successful putts indicating that motor performance was affected even whilst the outcome goal was achieved. These differences in movement kinematics used by the near elite golfers during fatigue suggest that the skilled behavior of expert performers might best be examined on an individual basis (Hammond, 2007; Park et al., 2015).

The findings of Mathers and Grealy (2014) raised the possibility that the same method of assessment could be used to investigate the effects of other psychological, physiological, or environmental variables that might impact on golf putting performance within individual expert golfers. Whilst demonstrating how a range of factors influence an individual will not generalize to the population as a whole, this method can provide considerable insights to the sports science and coaching communities who are committed to understanding the components of elite performance and developing individualized interventions (Yarrow et al., 2009). The ability to produce skilled performance within an environment of increased temperature would be a condition of relevance to golfers who play tournaments across the world and this was the subject of the present study.

The effects of increased temperature upon laboratory based motor tasks that require vigilance, reaction time, and temporal judgments have been fairly well documented, and can be attributed to either central (neural) influences, or motoric (peripheral) influences (Pilcher et al., 2002; Ross et al., 2006; Immink et al., 2012). A number of theories have been proposed to explain decrements in performance associated with thermal stress including the inverted U hypothesis and attentional changes (Hancock, 1986; Enander and Hygge, 1990; Ramsey, 1995; Vasmatzidis et al., 2002; Hancock and Vasmatzidis, 2003). Performance on vigilance tasks tends to decrease when subjects experience either an increase or decrease in core body temperature, although performance effects may also vary as a function of time, the complexity of the task, the direction of thermal change as well as the absolute value, and the level of skill of the participant groups (Hancock, 1986; Hancock and Vasmatzidis, 2003). However, whilst vigilance is undoubtedly a key element of many psychomotor skills in everyday life, it is not thought to be a critical sub-component of golf putting performance as the environmental features under consideration tend to be relatively stable from one moment to the next, and the ‘unexpected’ feature of vigilance tasks is not a characteristic of the putting skill. There has been similar interest in the impact of increased temperature on reaction time although again the results are probably less relevant to golf putting given the self-paced nature of the skill. Performance tracking on the other hand could be regarded as being particularly relevant to golf putting given the similarity between performance tracking and the on-line monitoring of the downswing movement of the golf putting stroke during the controlled act. In their review of the literature, Enander and Hygge (1990) noted that whilst generalizing across different study designs is problematic, there is evidence to suggest a decrement in tracking performance under cold conditions. Other research on the application of tracking skills, such as automotive driving performance where the driver is required to control the steering wheel on a predictable and visible path, has also found that different thermal conditions were associated with decrements in performance (Wyon et al., 1996). Theoretically at least, a skilled golfer might be subject to similar deleterious effects when aiming to control the movement of the putterhead during the putting skill.

Whilst the effects of temperature change on the *timing* of skilled motor actions such as golf putting have yet to be fully tested, some previous studies have tested the relationship between increased body temperature and the perception of time intervals in various laboratory tasks. Wearden and Penton-Voak (1995) explored the relationships between body temperature and the estimation and production of timed durations and concluded that humans use a temperature-sensitive mechanism to regulate their time judgments that speeds up when body temperature increases. However, the majority of the studies reviewed did not measure the individual’s thermoregulatory response to the heat application through core body measurement, known to be the preferred index of heat stress (see Hancock, 1986; Enander, 1989; Vasmatzidis et al., 2002, for a more detailed review) and, therefore, have limited applicability as a result. Hancock (1982) defined dynamic changes in core body temperature as changes that could not be compensated for through normal thermoregulation, and outlined the magnitude of change that would be required to generate statistically significant decrements in four cognitive tasks and general physiological tolerance. Hancock and Vasmatzidis (1998) suggested that whilst decrements in vigilance and dual task performance would be evident after relatively small rises in deep body temperature (0.06–0.22°C), more significant rises would be required before decrements on other cognitive tasks and physiological tolerance would be observed (i.e., 1.33–1.67°C). Whilst this research has shed some light on the impact of increasing temperature on cognitive function, there remains a need to investigate the extent to which the effects of increased temperature are translated to specific sub-components of precisely timed motor acts, such as golf putting, so that players and coaches can monitor and assess golf putting behaviors in a situation that relates to competitive play.

The current investigation centered on the impact of increased core body temperature upon golf putting performance and hypothesized that an increase in body temperature would speed up of the timing of the putting action (i.e., decrease the duration of the downswing) and, consequently, increase the putterhead velocity at ball impact. Such an increase could have a detrimental impact on putting outcome as it would result in the ball over-shooting the hole on longer putts, or cause the ball to travel with excessive velocity along a perceived line of putt (with a natural slope or borrow) and miss the cup. In relation to the model of motor timing proposed by Craig et al. (2000) it was also hypothesized that this thermal effect would be most apparent for golfers who used 1/*T*^2^ as part of the method for scaling their putting movement.

## Materials and Methods

### Participants

Six elite amateur golfers consisting of three males and three females (mean age = 20.67 years, *s* = 1.03 years) who were part of a University International Sports Scholarship Programme participated in the study. Four of the participants also took part in the fatigue study reported by Mathers and Grealy (2014). Local ethical approval was granted for this study and all participants gave written informed consent in accordance with the Declaration of Helsinki.

### Materials

The equipment and procedures for measuring the putting actions in this study were the same as those described in Mathers and Grealy (2014). The study was carried out in a purpose built indoor artificial putting green (measuring 2 m by 5 m) that had four standard golf holes (diameter 10.8 cm) embedded at distances of 1, 2, 3, and 4 m from defined start positions at one end of the structure. The surface was covered in a green colored synthetic textile material with similar retarding characteristics to putting surfaces used in elite competition (USGA Stimpmeter reading of 3.05 m). The movement of the putterhead was recorded using three Qualisys motion capture cameras sampling at 240 Hz with markers attached to the heel and toe of the putterhead. Each golfer used their own putter and supplied a golf ball that conformed to the cover type and compression of their choice during the period of data collection. Pre-putt core body temperature was measured by a Squirrel SQ400 data logger (Grant Instruments Ltd, Cambridge) where the miniature thermometer was placed within the ear canal of the participant. The data logger provided a continuous data stream that recorded core body temperature every 5 s and could be noted accurately at the moment of each putting trial (Fuller et al., 1999; Moran and Mendal, 2002).

### Procedure

Each participant performed 80 putting trials (20 trials for each of the four putt distances; 1, 2, 3, and 4 m) at normal body temperature and 80 putting trials when their core body temperature had been increased by approximately 1°C. Body temperature was increased using a steam pod (Portable Home Steam Sauna Pod, First Vitality International, UK) designed to apply heat to the body within the pod but not to the ambient air temperature within the laboratory, or the air that surrounded the participant’s head, which may have contaminated the temperature measurements. During the pilot phase, none of the participants reported that the experience of entering the steam pod (in the absence of thermal stress) caused discomfort, fatigue or provided a distraction to the putting task. For practical reasons each session recorded the putting performance at only one of the putt distances, and the order of putt distance was randomized between participants. It should also be noted that the participant group’s previous experience with the research equipment and high level of putting expertise suggested that any practice effects resulting from presentation order would be minimal. The participants began each data collection session with a 10-min familiarization period. A small sample of urine was tested using a litmus test to ensure that the participant was in a state of full hydration, and data from female participants was collected during the follicular phase of their menstrual cycle to control for hormonal temperature fluctuations that typically occur during ovulation (Shinohara et al., 2000). The Squirrel digital thermometer was then placed into the left ear canal of the participant to measure core body temperature and then secured in position by headphones packed with cotton wool. The participant then had a further familiarization period to ensure that the putting movement was not obstructed by the thermometer or headphones whilst the temperature reading increased from the ambient laboratory temperature (21°C) to the participant’s core temperature (typically 36.8°C). Core temperature was considered to be reached when the temperature reading had remained stable for a period of 10 min, after which the baseline data collection took place.

Each participant performed 20 putting trials at normal body temperature then removed their outer layers of clothing and entered the steam pod until their core body temperature had increased by about 1°C (or until the participant began to experience feelings of moderate discomfort), a process that typically took about 20 min. None of the participants reported feelings of discomfort before a core body temperature rise of 1°C had been reached. The participant then moved from the steam pod, dried any residual fluid and sweat then replaced their original layers of clothing before performing a further twenty putting trials to the same putt distance as their core temperature gradually returned to pre-treatment levels. To assess how close the execution of each putt was to ideal, participants were asked to rate the velocity of the ball as it entered the putting cup relative to their intended (ideal) velocity immediately after each trial. They did this using a line bisection method. Participants used a horizontal 10 cm line that was labeled as ‘ideal velocity’ at the midpoint (5 cm), ‘slower than ideal’ (0–5 cm) and ‘faster than ideal’ (5–10 cm) to place a vertical mark on the line at the point that represented the putt’s actual velocity. The velocities of the putterhead at impact on those trials where the participants had marked their performance as ideal were extracted and used to establish the ideal velocity for each putt distance. This method allowed for any individual differences in preferred goal behaviors to be established and for any changes in kinematic outcome to be assessed.

### Data Processing Methods

Kinematic data were analyzed using Labview software. First data were filtered using a Gaussian filter with a sigma value of six. Then the start and end of the downswing movement of the putting stroke, and the point of impact between the putterhead and the golf ball (determined from the calibration trials) were noted. The downswing data for each putt were used to determine: squared velocity of the putterhead at impact (*V*_c_^2^); proportion of time-to-impact from the start of the downswing (*P*_t_); the inverse of the downswing duration squared (1/*T*^2^); the amplitude of the downswing squared (*D*^2^); and the shape of the velocity profile of the movement (*k*) as defined by Craig et al. (2000). To determine the scaling strategies being used by each golfer multiple regression analyses were conducted where *P*_t_, 1/*T*^2^, *D*^2^ and *k* were entered as predictors of the squared putterhead velocity at ball impact (*V*_c_^2^). Examination of the data did not reveal any significant autocorrelations. Additionally, the collinearity amongst the predictor variables was examined for each participant. An analysis procedure was adopted that deleted any variables from the model that showed high bivariate correlations and Variance Inflation Factor values greater than three. This procedure was carried out for each golfer’s data but did not result in any variables being removed from the models.

## Results

A paired samples *t*-test confirmed a significant rise in core body temperature between the normal and elevated pre-putt temperature readings [*t*(5) = 12.88, *p* < 0.001]. On exiting the steam pod the participants’ core body temperature was close to 1°C above normal, and returned to normal over the course of the subsequent twenty putting trials. Thus, the overall mean increase in body temperature across the trials was 0.60°C (*s* = 0.16°C).

The number of successful putts at each body temperature was recorded and a paired samples *t*-test revealed that there was no difference in the percentage success rate between the normal temperature (mean value = 80.33%) and elevated temperature (mean value = 81.56%) conditions [*t*(5) = 0.34, *p* = 0.74]. Only successful putts were included in the inferential analyses described below.

**FIGURE 1:**
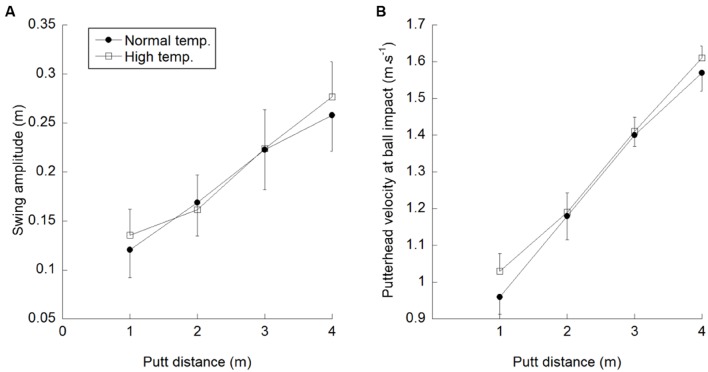
**Mean and standard deviations of **(A)** swing amplitude and **(B)** putterhead velocity at impact for the four putt distances under normal and elevated temperature conditions**.

It was hypothesized that if a rise in core body temperature sped up the timing mechanism responsible for motor control, then under high temperature conditions swing duration would decrease, swing amplitude would remain constant and the velocity of the putterhead would increase as a consequence. **Table 1** shows mean putterhead velocities across the four putt distances for each participant when their core body temperature was normal and elevated. A two-way (temperature × putt distance) repeated measures ANOVA on these data showed a significant temperature by distance interaction [*F*(3,15) = 4.55, *p* = 0.019, $\eta_{p}^{2}$ = 0.48]. A *post hoc* Tukey HSD test revealed that there was a significant difference in putterhead velocity between normal and elevated body temperatures for the putts made to 1 and 4 m (*p* = 0.05), but not for the distances of 2 and 3 m (see **Figure 1B**). As expected, the main effect of distance was significant [*F*(3,15) = 356.08, *p* < 0.001, $\eta_{p}^{2}$ = 0.98] and the results also showed a significant main effect of body temperature on putterhead velocity [*F*(1,5) = 235.80, *p* < 0.001, $\eta_{p}^{2}$ = 0.98], with an increase in velocity at ball impact when the body temperature was higher (mean value = 1.31 m s^-1^) compared to normal body temperature (mean value = 1.28 m s^-1^).

**Table 1:** Average putterhead velocity at ball impact (m s^-1^) for each of the putting distances at normal and elevated core body temperatures.

	1 m		2 m		3 m		4 m	
				
	Normal	High	Normal	High	Normal	High	Normal	High
P1	1.00	1.04	1.23	1.27	1.43	1.45	1.65	1.67
P2	1.01	1.10	1.25	1.21	1.41	1.44	1.57	1.62
P3	0.91	0.98	1.23	1.20	1.38	1.39	1.53	1.58
P4	0.98	1.06	1.13	1.11	1.36	1.35	1.52	1.57
P5	0.97	1.01	1.15	1.17	1.38	1.38	1.55	1.62
P6	0.90	0.98	1.10	1.18	1.43	1.41	1.61	1.62


To test the hypothesis that raised body temperature would have resulted in a decrease in swing duration (and thus an increase in putterhead velocity at impact) two two-way repeated measures ANOVAs [temperature (2) × putt distance (4)] were carried out with swing duration (*T*) and swing amplitude (*D*) as the dependent measures. For swing duration there was no significant main effect of temperature [*F*(1,5) = 0.39, *p* = 0.56, $\eta_{p}^{2}$ = 0.07], or putt distance [*F*(3,15) = 0.56, *p* = 0.65, $\eta_{p}^{2}$ = 0.10] or a significant interaction [*F*(3,15) = 1.11, *p* = 0.38, $\eta_{p}^{2}$ = 0.18]. When swing amplitude was the dependent measure the interaction between temperature and distance was significant [*F*(3,15) = 9.92, *p* = 0.001, $\eta_{p}^{2}$ = 0.67]. A *post hoc* Tukey test showed a significant increase in swing amplitude in the high temperature condition for putt distances of 1 and 4 m (*p* < 0.05) but not for the 2 and 3 m distances (see **Figure 1A**).

There was also a significant main effect of temperature [*F*(1,5) = 6.86, *p* = 0.047, $\eta_{p}^{2}$ = 0.56] with swing amplitudes being increased under high temperature (mean value = 0.20 m) compared to normal temperature (mean value = 0.19 m) conditions. The significant main effect of distance [*F*(3,15) = 54.49, *p* < 0.001, $\eta_{p}^{2}$ = 0.92] reflected a preference for increasing swing amplitude to achieve longer putt distances (see scaling methods below).

No clear predictions could be made about whether the proportion of the swing duration before the ball is struck would be influenced by an increase in body temperature, and a repeated measures ANOVAs (temperature × putt distance) showed no significant main effect of temperature [*F*(1,5) = 2.94, *p* = 0.15, $\eta_{p}^{2}$ = 0.37], or distance [*F*(3,15) = 0.86, *p* = 0.48, $\eta_{p}^{2}$ = 0.15] or a significant interaction [*F*(3,15) = 0.79, *p* = 0.52, $\eta_{p}^{2}$ = 0.14]. Differences between actual and ideal velocity (expressed as a percentage) at normal and elevated temperature were also examined. Separate two-way [temperature (2) × putt distance (4)] repeated measures ANOVAs were then conducted with mean difference and mean RMS difference as dependent variables. There was a significant temperature by putt distance interaction for mean differences and a *post hoc* Tukey HSD test showed that at a distance of 1 m there were significantly greater mean difference when body temperature was normal (mean value = 5.37%) compared to the mean difference when body temperature was elevated (mean value = 0.34%, *p* = 0.05). Neither of the ANOVAs showed significant main effects for temperature [mean difference; *F*(1,5) = 1.14, *p* = 0.33, mean RMS difference; *F*(1,5) = 0.57, *p* = 0.49]. There were no significant differences between the two temperature states for the other putt distances.

Finally, multiple regression analyses were carried out to determine the extent to which changes in *P*_t_, 1/*T*^2^, *D*^2^, and *k* predicted squared putterhead velocity at impact at normal and increased temperature for each participant. The results of these analyses are summarized in **Table 2.** Three of the participants showed changes in their scaling models between the two temperature conditions but the other three did not. Two of the participants who showed changes (P2 and P4) had a simpler scaling model when their temperature was raised, whilst P6’s scaling model changed from *D*^2^, 1/*T*^2^, and *P*_t_, under normal temperature conditions to *D*^2^, 1/*T*^2^, and *k* under elevated temperature conditions.

**Table 2:** Outcomes of multiple regression analyses showing the models used to scale the velocity at impact during baseline and increased temperature.

			Standardized β			
					
	Model	Adjusted *r^2^*	*D^2^*	*1/T^2^*	*P_t_*	*k*
**Normal Temperature**				
P1	*p* < 0.001	0.966	1.11^∗∗^	0.51^∗∗^	-0.43^∗∗^	0.05
P2	*p* < 0.001	0.941	0.93^∗∗^	0.21^∗^	-0.13^∗^	0.04
P3	*p* < 0.001	0.940	1.00^∗∗^	0.28^∗∗^	-0.14^∗∗^	0.02
P4	*p* < 0.001	0.965	1.02^∗∗^	0.18^∗∗^	-0.07^∗^	-0.01
P5	*p* < 0.001	0.912	0.87^∗∗^	0.51^∗∗^	-0.21^∗∗^	-0.08
P6	*p* < 0.001	0.976	0.98^∗∗^	0.28^∗∗^	-0.14^∗∗^	0.02
**Elevated temperature**				
P1	*p* < 0.001	0.956	1.08^∗∗^	0.34^∗∗^	-0.025^∗∗^	0.04
P2	*p* < 0.001	0.959	0.99^∗∗^	0.18^∗∗^	-0.13	0.01
P3	*p* < 0.001	0.914	0.80^∗∗^	0.19^∗∗^	-0.150^∗∗^	-0.02
P4	*p* < 0.001	0.935	0.98^∗∗^	0.22^∗∗^	0.02	-0.05
P5	*p* < 0.001	0.925	0.80^∗∗^	0.50^∗∗^	-0.35^∗∗^	0.03
P6	*p* < 0.001	0.926	1.07^∗∗^	0.29^∗∗^	-0.03	0.11^∗^


*Participants P1, P3, P4, and P6 also participated in the fatigue study reported by Mathers and Grealy (2014). ^∗^p < 0.05 and ^∗∗^p < 0.001.*

## Discussion

The results revealed no significant difference in the number of putts that were successfully holed between the normal temperature and elevated temperature. Whilst this finding could be viewed as somewhat surprising given the evidence that exists to link elevated core temperatures to decreased performance in motor tasks (Enander and Hygge, 1990; Pilcher et al., 2002; Hancock and Vasmatzidis, 2003) it should be acknowledged that the ‘number of putts holed’ is a rather insensitive outcome measure and not the central focus of the investigation. The putting task was carried out on a flat and predictable surface where success rested simply on the participant’s ability to align the putterhead toward the center of the hole and generate a golf ball velocity and direction that would allow the golf ball to enter the putting cup. These requirements were well within the capability of this elite group (especially for the 1 m putt) as even within the heightened thermic conditions less than 1% of the trials failed to reach the putting cup, or were struck with excessive force that forced the ball to travel directly over the putting cup. Instead, the experimental task was used to create an ecologically valid framework to explore the kinematic control of the putterhead during the skilled act at normal and at increased core body temperature.

Increasing body temperature had some interesting and unexpected effects on the kinematics of the putting actions of the elite golfers when the ball was ‘holed’. Contrary to our prediction, the analyses showed that increasing body temperature did not significantly change swing duration, or speed up the timing of the putt. However, swing amplitude did increase for the 1 and 4 m putts when body temperature was raised, and a corresponding increase in putterhead velocity at impact was also seen for these two putting distances. These findings are not in line with the previous work that suggests humans use a temperature-sensitive mechanism to regulate their temporal judgments for time estimation and for production of a time-sensitive motor task (Wearden and Penton-Voak, 1995). It is not clear why only the 1 and 4 m putt distances were affected by the rise in temperature, however, it is possible that increased elevated body temperature encouraged a change in attentional focus and performance strategy for these specific lengths of putt that were translated to the movement kinematics used to carry out the task. Perhaps the task of holing a 1 m putt on a level surface under normal temperature conditions may have been insufficient to encourage these elite players to enter their individual zones of optimal functioning (Jokela and Hanin, 1999). The results of the mean difference from ideal velocity and mean RMS velocity difference suggest that the players probably chose a different strategy to hole the 1 m putt at an elevated core temperature, and that deviations from ideal velocity were significantly lower on the 1m putt distance at increased temperature when compared to the pre-treatment phase. The increase in temperature may have encouraged them to purposefully strike the ball with a slightly greater velocity (i.e., to be more positive) and increase their intensity and effort in a similar way to that shown by Aune et al. (2008) in their study on the effects of fatigue. During the 4 m putt, where an unsuccessful outcome is more likely (Pelz, 2000), the players may simply have decided to focus their attention on the process goal (i.e., making a smooth stroke) and prioritize the duration of the stroke at the expense of other control variables. During the 2 and 3 m putts where the outcome is less certain, the specific challenge of intermediate difficulty may have created a more task-relevant focus that preserved the control parameters in their natural form as suggested by Nideffer (1976), Beilock et al. (2001) and Perkins-Ceccato et al. (2003). Changes in strategy that result from increased stress associated with fatigue have been noted previously (Matthews and Desmond, 2002) and other authors have noted the ability of elite performers to modify successfully various control parameters when required (Dal Monte et al., 2002; Knight, 2004). Whilst these explanations may be plausible for this particular group, future work should aim to explore any changes in the kinematic patterns involved for specific putt distances and with a larger elite population. Clarity might also be sought on the extent to which the participants may have consciously changed the kinematics of the stroke to accommodate the increase in body temperature.

Although swing amplitude was by far the most dominant predictor of performance scaling for all of the golfers, some interesting changes in the methods used to scale the putts between the baseline and at increased temperature were noted. Two participants used a simplified method involving fewer predictors when temperature was raised, whilst one (P6) changed their scaling model from *D*^2^, 1/*T*^2^ and *P*_t_, under normal temperature conditions to *D*^2^, 1/*T*^2^, and *k* under elevated temperature conditions. The other three golfers retained the same kinematic pattern across the two experimental conditions. Changes in movement kinematics that result from increased fatigue, as opposed to heat, have been noted previously (Mathers and Grealy, 2014) and this phenomenon has also been noted by Aune et al. (2008) who discussed the ability of expert performers to utilize various compensation strategies in times of stress.

Whilst the results of this laboratory-based experiment showed changes in movement kinematics occurred with increased body temperature, these were not sufficient to affect the success rates for this participant group. However, the kinematic changes observed here could conceivably decrease putting outcome in field-based putting tasks where the task demands are considerably more complex. The velocity of the ball becomes a more critical element of success when a golfer has to perceive a putt distance and green characteristics, choose a particular putting strategy then translate this perception into an action (Pelz, 2000). Moreover, if increased temperature encourages golfers to focus their attention on the alignment and the velocity of the putterhead at impact, this may well be at the expense of other performance sub-components that are intrinsic aspects of the skill of golf putting such as perceiving the environment (Penner, 2002; Hancock and Vasmatzidis, 2003; Hume et al., 2005) or deciding on a preferred strategy (Pelz, 2000). Such attentional changes in the real life setting may reduce the number of successful putts on the golf course (Wulf, 2013).

The results revealed no consistent changes in the scaling models that were used to control the velocity of the putterhead at impact, although during increased temperature there were some differences in the number of control parameters used by two of the players. This finding bears some similarity to a previous experiment that revealed some reduction in the number of control parameters used to scale the putting action during a condition of fatigue (Mathers and Grealy, 2014). Perhaps future experiments should aim to establish the specific impact of hot climates on individual core temperature and measure the impact on more wide-ranging performance sub-components when the core body temperature is moving away from the baseline as well as when it is returning to the pre-treatment state. Experiments might also examine the effect of increased temperature during elite competition when anxiety and fatigue may also impact on golf putting performance. Perhaps too, the performance of elite or expert performers might be examined on an individual basis, using sensitive performance measures and protocols that provide a better understanding of the basic control mechanisms that underlie sports behavior (Park et al., 2015).

## Conclusion

The results from this experiment showed a significant main effect of body temperature on putterhead velocity at ball impact at 1 and 4 m putt distances, with an increase in velocity being produced when body temperature was higher than normal. The results also showed whilst the increase in temperature altered the motor control patterns used by these golfers there was no significant difference in the success rate between baseline and temperature conditions for these laboratory-based putting tasks. Golfers who are susceptible to the effects of increased temperature should ensure that they select a strategy for holing out that accounts for a modest increase in the velocity of the putterhead during the impact with the golf ball. These findings may provide some general guidelines for the competitive golfer, and add weight to the argument that the performance of more expert performers should be examined on an individual basis and with sensitive methods of kinematic analysis that go far beyond the basic outcome measures that have prevailed thus far.

## Author Contributions

JM and MG were responsible for the concept and design of the experiment, the interpretation of data, the drafting and revising the work critically, approving the final version and agree to be accountable for the work.

## Conflict of Interest Statement

The authors declare that the research was conducted in the absence of any commercial or financial relationships that could be construed as a potential conflict of interest.
